# Plasma Angiotensin Converting Enzyme 2 (ACE2) Activity in Healthy Controls and Patients with Cardiovascular Risk Factors and/or Disease

**DOI:** 10.3390/jpm12091495

**Published:** 2022-09-13

**Authors:** Hui Yin Lim, Sheila K. Patel, Ping Huang, Mark Tacey, Kay Weng Choy, Julie Wang, Geoffrey Donnan, Harshal H. Nandurkar, Prahlad Ho, Louise M. Burrell

**Affiliations:** 1 Northern Pathology Victoria, Northern Health, Epping, Melbourne, VIC 3076, Australia; 2 Department of Medicine, Northern Health, University of Melbourne, Epping, Melbourne, VIC 3076, Australia; 3 Department of Medicine, Austin Health, University of Melbourne, Heidelberg, Melbourne, VIC 3084, Australia; 4 Australian Centre for Blood Diseases, Monash University, Melbourne VIC 3004, Australia; 5 The Northern Hospital, Epping, VIC 3076, Australia; 6 Department of Medicine & Radiology, University of Melbourne, Parkville, VIC 3052, Australia; 7 The Melbourne Brain Centre, Royal Melbourne Hospital, University of Melbourne, Parkville, VIC 3052, Australia

**Keywords:** angiotensin converting enzyme 2, coagulation, cardiovascular disease, renin angiotensin system

## Abstract

Angiotensin converting enzyme 2 (ACE2) is an endogenous negative regulator of the renin-angiotensin system, a key factor in the development of cardiovascular disease (CVD). ACE2 is also used by SARS-CoV-2 for host cell entry. Given that COVID-19 is associated with hypercoagulability, it is timely to explore the potential relationship between plasma ACE2 activity and the coagulation profile. In this cross-sectional study, ACE2 activity and global coagulation assays (GCA) including thromboelastography, thrombin, and fibrin generation were measured in adult healthy controls (*n* = 123; mean age 41 ± 17 years; 35% male) and in patients with cardiovascular risk factors and/or disease (*n* = 258; mean age 65 ± 14 years; 55% male). ACE2 activity was significantly lower in controls compared to patients with cardiovascular risk factors and/or disease (median 0.10 (0.02, 3.33) vs. 5.99 (1.95, 10.37) pmol/mL/min, *p* < 0.001). Of the healthy controls, 48% had undetectable ACE2 activity. Controls with detectable ACE2 had lower maximum amplitude (*p* < 0.001). In patients with cardiovascular risk factors and/or disease, those in the 3rd tertile were older and male (*p* = 0.002), with a higher Framingham grade and increased number of cardiovascular risk factors (*p* < 0.001). In conclusion, plasma ACE2 activity is undetectable to very low in young healthy controls with minimal clinically relevant associations to GCA. Patients with cardiovascular risk factors and/or disease have increased plasma ACE2 activity, suggesting that it may be an important biomarker of endothelial dysfunction and atherosclerosis.

## 1. Introduction

Activation of the renin-angiotensin system (RAS) is a key factor in the pathophysiology of cardiovascular disease (CVD) [1,2,3]. Angiotensin converting enzyme 2 (ACE2) is an endogenous negative regulator of the RAS through its action to degrade angiotensin II [2]. ACE2 is present on vascular endothelial cells and proteolytic shedding results in a soluble form that can be measured in plasma. There are very few studies of plasma ACE2 levels in healthy individuals, but in most studies, levels are from undetectable to very low [1,4,5]. This contrasts with levels in patients with cardiovascular risk factors or disease, where circulating ACE2 is increased and associated with adverse outcomes [3,6,7,8,9,10,11,12,13,14].

There has been renewed interest in an improved understanding of the role of ACE2 since the discovery that ACE2 is used by the acute respiratory syndrome-coronavirus-2 (SARS-CoV-2) for host cell entry [15]. We recently reported that patients with COVID-19 have increased circulating ACE2 levels compared to non-COVID controls [4], and that these changes persist for at least 8 months [5]. Given that COVID-19 is also associated with a hypercoagulable state [16,17,18], it is timely to explore the potential relationship between plasma ACE2 activity and the coagulation profile.

Our group has an interest in predictors of hypercoagulability [19,20]. Routine coagulation tests such as activated partial thromboplastin time (APTT) and prothrombin time (PT) only estimate the starting time of clot formation, representing only 5% of thrombin generation, are not representative of total clot formation [21] and hence are poor predictors of thrombotic risk. Global coagulation assays such as thrombin generation, thromboelastography and fibrin generation provide a more comprehensive assessment of hypercoagulability [20,22].

Critical to further our ability in predicting thrombotic and cardiovascular risk, particularly in the COVID-19 population, we first need to understand the differences seen in the healthy control population and explore the interplay of ACE2 with the coagulation system. This study was conducted in people without COVID-19 and was designed to evaluate plasma ACE2 activity in healthy controls in comparison with patients with cardiovascular risk factors and/or disease, and the potential impact of plasma ACE2 activity levels on global coagulation assay parameters.

## 2. Materials and Methods

This is a cross-sectional study involving two parallel cohorts of study populations in the outpatient setting: healthy controls and patients with cardiovascular risk factors and/or disease. Healthy controls aged 18 to 80 years old, with no self-reported history of thrombosis or CVD, were recruited. Those on anticoagulants, anti-platelet, hormonal therapy or with cardiovascular risk factors requiring active medical management were excluded. This study was approved by the Austin and Northern Health Human Research Ethics Committees (H2013/04977 and P5/13). A cohort of adult patients with known cardiovascular risk factors and/or disease on active treatment including hypertension, hyperlipidaemia and diabetes as well as smoking, male gender, age >65 years and chronic kidney disease (defined as estimated glomerular filtration rate of <30 mL/min/1.73 m^3^) were also recruited. A history of cardiovascular disease (coronary heart disease, peripheral arterial disease, and cerebrovascular disease) was annotated where present. Patients on anticoagulation or with active infection, cirrhosis or malignancy were excluded. This study was approved by the Austin Health Human Research Ethics Committee (HREC/Austin/16/459). Written informed consent was obtained from all participants.

In both cohorts, a full blood count, renal and liver function tests, coagulation studies and von Willebrand studies were performed by an accredited laboratory as per manufacturers’ guidelines. Specific analyses of cardiovascular risk markers, such as glycated haemoglobin (HbA1c) and lipid studies, were also performed. Additional citrated blood samples were tested immediately with thromboelastography (TEG) and the remainder double-spun at 2500 g to obtain platelet-poor plasma. The platelet-poor plasma was stored at −80 °C for the measurement of plasma ACE2 activity, as well as thrombin generation using calibrated automated thrombogram (CAT), fibrin generation using overall haemostatic potential (OHP) and tissue factor pathway inhibitor (TFPI).

*Plasma ACE2 activity:* The catalytic activity of ACE2 was measured using a validated, sensitive quenched fluorescent substrate-based assay as previously described [1]. A volume of 250 µL of thawed platelet-poor plasma was diluted into low-ionic-strength buffer (20 mmol/L Tris-HCl, pH 6.5) and added to 200 μL ANXSepharose 4 Fast-Flow resin (Amersham Biosciences, GE Healthcare, Uppsala, Sweden) to remove an endogenous inhibitor of ACE2 activity [1]. After binding and washing, the resulting eluate was assayed for ACE2 catalytic activity. Duplicate samples were incubated with the ACE2-specific quenched fluorescent substrate, with or without 100 mM ethylenediaminetetraacetic acid. The rate of substrate cleavage was determined by comparison to a standard curve of the free fluorophore, 4-amino-methoxycoumarin (MCA; Sigma, MO, USA) and expressed as pmole of substrate cleaved/mL of plasma/min. The intra-assay and inter-assay coefficients of variation were 5.6% and 11.8%, respectively. The limit of the detection for the assay is 0.04 pmol/mL/min and undetectable ACE2 samples were given a value of half the detection limit (i.e., 0.02) for the purpose of statistical analysis.

*Thromboelastography (TEG^®^)*: All samples were tested using citrated kaolin assay on TEG 5000^®^ (Hemonetics, Boston, MA, USA) within 4 h of collection. 1 mL of whole citrate blood was added to a kaolin tube and 340 μL of this sample was then pipetted into the cup containing 20 μL of 0.2M calcium chloride and the TEG^®^ assay immediately ran. Routine parameters included R-time (time to clot onset), maximum amplitude (strength of fibrin clot, MA), α-angle (rate of clot formation), and clot lysis (rate of clot breakdown, LY30).

*Calibrated automated thrombogram (CAT):* The rate and extent of thrombin generated in PPP after a 5 pM tissue factor stimulus, were measured using CAT (Diagnostica STAGO, Maastricht, The Netherlands). A volume of 80 μL of thawed PPP was added to either 20 µL of PPP (low) reagent or 20 µL of thrombin calibrator and incubated at 37 °C. The mixture of fluorescence buffer and substrate is then dispensed into each well via the automated fluorometer. A dedicated software program, Thrombinoscope BV, is used to calculate thrombin activity against the calibrator and reports parameters including lag time (time to start of clot formation in minutes), endogenous thrombin potential (ETP), area under the curve representing the amount of thrombin generated (nM/min), peak (maximal point of thrombin generation, nM), time to peak (minutes), and velocity index (rate of thrombin generation, nM/min) [23].

*Overall haemostatic potential (OHP) assay*: This is a spectrophotometric assessment of fibrin-aggregation over 60 min in thawed PPP using the FLUOstar^®^ Optima (BMG Labtech, Offenburg, Germany) plate reader at 405 nM and 37 °C. For each sample, there is a corresponding overall coagulation potential (OCP) buffer of Tris/NaCl/CaCl2/thrombin (final concentration 66 nM Tris, 130 mM NaCl, 35 mL CaCl_2_, thrombin 0.006 IU/mL; pH 7.0) and an overall haemostatic potential (OHP) buffer of Tris/NaCl/ CaCl2/thrombin/tissue-plasminogen activator (600 ng/mL). The difference between the areas under the two curves reflects the overall fibrinolysis potential (OFP). All samples were performed in triplicate.

*Tissue factor pathway inhibitor (TFPI*): The Thermo Scientific^TM^ Human TFPI ELISA (enzyme-linked immunosorbent assay) kit (Thermo Fisher, Waltham, MA, USA) was used to measure the total TFPI level in PPP using the FLUOstar^®^ Optima (BMG Labtech) plate reader. All samples were performed in duplicates. A target-specific antibody was pre-coated in the wells of the microplate. Samples were then added into these wells and bound to the capture antibody. The sandwich was formed by the addition of a second antibody followed by a substrate solution that reacts with the antibody-target complex to produce a signal measured at 450 nM. The intensity of this signal is directly proportional to the concentration of the TFPI.

*Statistical analysis:* Data were collected using Microsoft Excel with statistical analysis performed using Stata version 15.1 (StataCorp, College Station, TX, USA). Comparisons were conducted using t-tests for normally distributed variables, and Mann–Whitney tests for those variables found to be non-normally distributed. Chi-squared tests were used to analyse categorical variables. Pearson and Spearman’s correlation coefficients were calculated for the comparison of selected continuous variables. With the majority of variables being normally distributed, multivariable linear regression analysis was conducted to identify differences between the assay parameters, adjusting for the effect of age and gender. Statistical significance was set at *p* < 0.05.

## 3. Results

### 3.1. Healthy Controls

Plasma ACE2 activity was measured in 123 healthy controls (mean age 41 years; 98 females (64%)). Male controls had higher ACE2 levels compared to females (median 2.18 (IQR 0.02, 5.34) vs. 0.02 (0.02, 2.08) pmol/mL/min, *p* = 0.014). The cohort was then split according to detectable (52%) or undetectable ACE2 activity (Table 1). There were no differences in terms of age or gender and no clinically significant differences on routine blood investigations between the two groups. With regard to the coagulation assays, controls with detectable plasma ACE2 activity had lower prothrombin time (PT) and D-dimer, although the levels were within normal reference interval. These controls also demonstrated maximum amplitude (clot strength) on thromboelastography (mean 57.4 vs. 62.0 mm, *p* < 0.001) with no significant differences seen on thrombin generation, fibrin generation or TFPI.

### 3.2. Patients with Cardiovascular Risk Factors and/or Disease Compared to Healthy Controls

**Figure 1:**
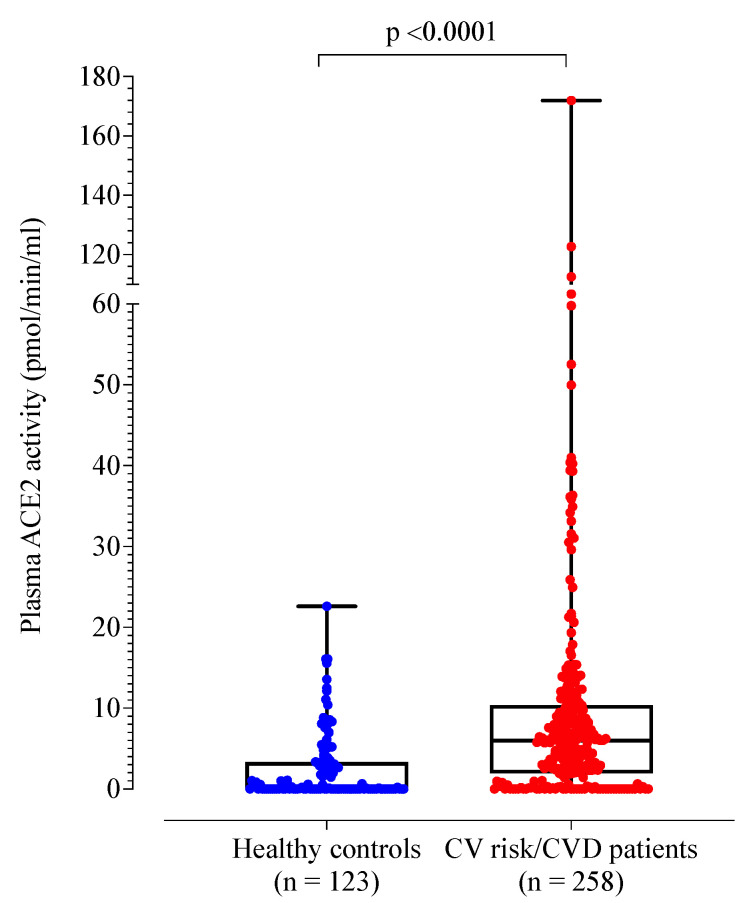
Box plot of plasma ACE2 activity levels of healthy controls compared to patients with cardiovascular risk factors and/or disease.

A total of 258 patients with cardiovascular risk factors and/or disease were recruited (mean age 65 years; 118 females (45%)). Compared to healthy controls, the patient cohort was older (*p* < 0.001) and more likely to be male (55% vs. 35%, *p* < 0.001) (Table 2). Plasma ACE2 activity was significantly higher in the patient cohort compared to healthy controls (Table 2 and Figure 1). By definition, the control group had no cardiovascular risk factors or disease. When compared to healthy controls, the patient cohort demonstrated higher D-dimer, fibrinogen, von Willebrand factor antigen and factor VIII levels (*p* < 0.001).

In the patient cohort, 53% were obese, 80% had hypertension, 60% had diabetes, 6% had a past history of stroke, 7% had peripheral vascular disease, 28% had coronary artery disease, and 17% were active smokers. The average number of cardiovascular risk factors was 4.5 (± 1.5) with statins prescribed in 66%, aspirin in 41%, and RAS blockers in 55% of cases. As expected, those with cardiovascular risk factors and/or disease had abnormalities on routine blood investigations in keeping with their risk profile including reduced eGFR and elevated HbA1c (Table 2).

The patient cohort also demonstrated more hypercoagulable global coagulation assay parameters including higher maximum amplitude (clot strength) on TEG^®^ and fibrin generation parameters (OCP and OHP) with lower fibrinolytic potential (OFP). Despite comparable CAT parameters, the patient cohort had significantly higher TFPI levels compared to the healthy controls.

### 3.3. Analysis of Patients with Cardiovascular Risk Factors and/or Disease according to Tertiles

We next analysed results according to tertiles of plasma ACE2 activity (Table 3). Those in the highest tertile of ACE2 activity (≥8.3 pmol/mL/min) were significantly older and more likely to be male, and a higher Framingham heart score (FHS) (*p* < 0.001). There was also increased risk of coronary artery disease (*p* = 0.012) which remained significant after adjustment for age and gender (*p* = 0.005). Plasma ACE2 activity levels did not differ according to body mass index, hypertension, diabetes, smoking status, kidney function, or medications including statins and RAS blockers. Routine blood investigations revealed no clinically significant differences across the ACE2 tertiles.

The results of the global coagulation assays showed that patients in the highest plasma ACE2 activity tertile had a lower maximum amplitude on thromboelastography (*p* = 0.009), which remained significant after adjustment for age and gender (*p* = 0.043) (Table 4). No differences were seen in thrombin generation. Fibrin generation measured using OCP was reduced in those with higher plasma ACE2 activity level although the overall fibrinolytic potential was preserved (*p* = 0.95). TFPI varied significantly across the tertiles and remained significant after adjustment for age and gender (*p* = 0.044).

## 4. Discussions

Our results showed that plasma ACE2 activity is undetectable to very low in healthy young individuals. This adds to the literature on ACE2 in healthy individuals and extends that work by reporting on ACE2 in people with no cardiovascular risk factors or disease on no medications. Previous studies of plasma ACE2 in “controls” included people with cardiovascular risk factors and/or comorbidities, who may be taking medications [4,13,24]. The observation that plasma ACE2 is higher in male controls compared to female controls (median 2.18 vs. 0.02 pmol/mL/min, *p* = 0.014) has been reported in previous studies [13] as well as in patients with COVID-19 [4]. Within the healthy controls, age did not influence plasma ACE2 activity level.

While there were statistically significant differences seen in several basic biochemical conventional coagulation studies including white cell count, platelet count, prothrombin time and D-dimer when comparing controls with undetectable ACE2 activity level and those detectable, the levels in both groups were well within normal clinical reference interval. We do note that there are subtle differences in the global coagulation assays in controls with detectable plasma ACE2 activity compared to those without, as seen with the lower maximum amplitude (clot strength) on thromboelastography (57.6 vs. 62.0 mm, *p* < 0.001) although the significance of this finding in a healthy population is unclear and warrants further investigations. We have previously shown that there is a complex interaction between global coagulation assays and cardiovascular risk factors in healthy controls, particularly with lower thrombin generation seen in these individuals [25]. 

Importantly, we found that plasma ACE2 activity is significantly increased in patients with cardiovascular risk factors and/or disease compared to healthy controls (Table 2) although there is minimal association with the coagulation profile. The findings of the higher ACE2 in patients with cardiovascular risk factors and/or disease are in keeping with previous studies [3,6,7,8,9,10,11,12,13,14,26]. In the patient cohort, plasma ACE2 activity levels did not differ according to body mass index, hypertension, diabetes, smoking status, kidney function, or medications including statins and RAS blockers (Table 3). Despite previous studies showing the role of cholesterol metabolism in the initiation of cardiovascular disease, especially the role of Soat1 (sterol O-acyltransferase)-mediated process [27,28,29], there were no differences seen between ACE2 activity levels and total cholesterol in this study. This may, in part, be explained by the use of statins across the different tertiles and highlights the potential of ACE2 activity as an independent risk factor. Narula et al. ranked ACE2 as the strongest predictor of death and superseded several clinical risk factors as a predictor of cardiovascular disease [13]. We did find that patients in the highest tertile were significantly older and more likely to be male. They were also more likely to have coronary artery disease (*p* = 0.012), a finding that remained significant after adjustment for age and gender (*p* = 0.005). These results suggest these are high risk patients, given we previously reported that increased plasma ACE2 activity independently increased the hazard of adverse long-term cardiovascular outcomes in patients with obstructive coronary artery disease [14].

Compared to healthy controls, patients with cardiovascular risk factors and/or disease had raised factor VIII and von Willebrand Factor antigen which play important roles in platelet adhesion and aggregation at sites of high shear stress. Importantly, von Willebrand factor has been shown to be an independent predictor of adverse cardiac events [30]. While inflammation has previously been reported to affect the stability of the coronary artery endothelium and plays an important link in the initiation of early atherosclerosis and downstream cardiovascular disease manifestations [31,32], this study showed minimal changes in C-reactive protein levels according to ACE2 activity tertiles. One postulation is that ACE2 dominates the impairment of the vascular endothelial function, leading to greater arterial stiffness via its detrimental effects on the RAS system balance. Hence, ACE2 likely fits in the upstream of the inflammation mechanism as an independent risk factor of the pathogenesis and early origin of atherosclerotic cardiovascular disease.

Patients with cardiovascular risk factors and/or disease also had hypercoagulable global coagulation parameters including increased maximum amplitude (indicating increased clot strength) and reduced clot lysis on TEG^®^, increased fibrin generation (OCP and OHP) with reduced overall fibrinolytic potential, as well as markedly increased TFPI levels. We have also previously shown that global coagulation assays have the potential to be biomarkers of cardiovascular disease [19,20], and considering the importance of Virchow’s triad, a triad of flow stasis, hypercoagulability and endothelial dysfunction, in the development of thrombosis, further studies looking at the combination of global coagulation assays and endothelial biomarkers such as ACE2 to predict future cardiovascular outcomes are underway [33].

When comparing results across the ACE2 activity tertiles in the patient cohort, those in the highest tertile had a reduced maximum amplitude on whole blood thromboelastography which persisted after adjustment, although a mean difference of 2–3 mm is unlikely to be of clinical significance. No significant differences were seen in thrombin and fibrin generation after adjustment for age and gender. There have been mixed findings in the literature pertaining to the role of thrombin generation in CVD including the LURIC study, which observed an inverse association between thrombin generation and CVD risk [34]. TFPI, an anticoagulant molecule, varied across the tertiles with highest values in the 1st and 3rd tertile, an effect that remained significant after adjustment for age and gender (*p* = 0.044). Increased TFPI has previously been associated with atherothrombotic disease [35], in keeping with the patients with the highest ACE2 activity levels demonstrating high Framingham grade [36] and increased number of cardiovascular risk factors.

One of the challenging concepts in understanding the role of ACE2 is that while tissue ACE2 is a negative regulator of RAS, and thus a protective marker against thrombosis and atherosclerosis, high risk patients have demonstrated conversely higher circulating ACE2 levels [3,6]. It is important to note that ACE2 is an integral membrane protein and exists in both a membrane bound and a soluble form; the latter results from proteolytic cleavage of the ectodomain by the proteinase ADAM17 (a disintegrin and metalloproteinase) [37]. In a recent study in which we measured both circulating and tissue levels of ACE2 in humans [26], we reported that reduced tissue levels were associated with elevated plasma ACE2 activity and that those with the highest plasma ACE2 activity had reduced tissue ACE2 expression and more severe myocardial fibrosis, an important predictor of death. These results in humans along with data from experimental studies [3,6,7,8,9,10,11,12,13,14,26,38] suggest that relative ACE2 tissue deficiency due to shedding into the circulation is responsible for tissue injury.

Overall, this paper highlights the complex interplay within the Virchow’s triad. RAS is vital in maintaining normal cardiovascular function, and a disruption to the balance within RAS can lead to a spectrum of cardiovascular diseases [39]. The important counter-regulatory role of ACE2 in atherosclerosis has been demonstrated in experimental studies which have shown that ACE2 overexpression promotes atherosclerotic plaque stability and attenuates atherosclerotic lesions [40,41,42] and that ACE2 deficiency is associated with accelerated atherosclerosis in mice [38]. The RAS is also known to have a direct impact on the coagulation system with an activated RAS system being known to increase platelet activation [43]. Interestingly, in this study, no significant coagulation changes were observed in what is meant to be a prothrombotic population, and TFPI—an anticoagulant— factor was increased. In addition to its role in CVD, ACE2 has also been identified as the entry point into cells for some coronaviruses including SARS-CoV-2 implicated in the current global pandemic [44]. SARS-CoV-2 infection downregulates ACE2 expression on cells thus preventing it from performing its normal function leading to increased availability of pro-inflammatory Ang II which propagates further tissue injury [45]. Regardless, these findings highlight the many unknowns and conundrums of the ACE2 function in both physiological and pathological states and warrants further investigation.

We acknowledge the small numbers in both study cohorts and the significant age and gender differences. We employed relatively strict criteria when defining our healthy controls. Another limitation of this study is that the use of angiotensin receptor/neprilysin inhibitors or mineralcorticoid receptor antagonists was not recorded. Nevertheless, to the best of our knowledge, this is the first study in which ACE 2 activity level and global coagulation assays that have been concurrently performed in both healthy control and patient cohorts. In most studies, subjects with disease are studied, whereas we have also examined healthy controls. It is important to understand the variability of an assay in a healthy population before extrapolating it to a population with risk factors or disease. Given the complexities of the ACE2 role, it is important to investigate how ACE2 interplays with the coagulation system in healthy controls and in patients with cardiovascular risk factors and/or disease in order to further our understanding of the role of ACE2 in thrombosis and COVID-19.

## 5. Conclusions

In summary, our study has demonstrated that ACE 2 activity, a negative regulator of the RAS system, is associated with increased cardiovascular risk factors and/or disease with minimal impact on coagulation parameters. In our ongoing endeavour to refine cardiovascular and thrombotic risk profiling, further large clinical studies are warranted to further understand the relationship between ACE2 and global coagulation assays.

## Figures and Tables

**Table 1:** Plasma ACE2 activity levels in healthy controls, categorized as undetectable and detectable levels.

	All Controls	Undetectable Plasma ACE2 Activity	Detectable Plasma ACE2 Activity	*p*-Value
N	123	59 (48.0%)	64 (52.0%)	
Age, mean (SD)	41 (17)	40 (17)	43 (17)	0.40
Sex (male)	43 (35.0%)	43 (72.9%)	43 (67.2%)	0.29
**Plasma ACE2 activity** (pmol/mL/min)	0.10 (0.02, 3.33)	0.02	3.20 (1.17, 7.82)	
**Baseline investigations**				
Haemoglobin (g/dL)	142.0 (136.0, 153.0)	141.0 (135.0, 153.0)	142.0 (137.0, 153.0)	0.38
White cell count (×10^9^/L)	5.7 (5.0, 6.6)	6.1 (5.3, 6.8)	5.6 (4.8, 6.3)	**0.031**
Platelets (×10^9^/L)	245.0 (215.0, 279.0)	257.0 (229.0, 293.0)	230.0 (202.0, 272.0)	**0.006**
Prothrombin time (sec)	11.0 (10.3, 12.6)	12.2 (11.0, 13.2)	10.9 (10.1, 11.0)	**<0.001**
APTT (sec)	28.0 (26.0, 31.0)	28.8 (26.4, 31.6)	27.9 (25.0, 30.2)	0.062
Fibrinogen (g/L)	3.0 (2.5, 3.5)	3.1 (2.6, 3.5)	2.9 (2.5, 3.5)	0.12
D-dimer (mg/L FEU)	270.0 (150.0, 290.0)	270.0 (190.0, 300.0)	190.0 (150.0, 270.0)	**0.035**
Factor VIII level (%)	104.0 (86.0, 145.0)	111.0 (86.0, 151.0)	102.0 (84.0, 130.5)	0.098
Von Willebrand factor antigen (%)	100.0 (86.0, 127.0)	141.0 (135.0, 153.0)	142.0 (137.0, 153.0)	0.38
eGFR (mL/min/1.73 m^2^)	99.5 (90.0, 116.0)	103.0 (89.0, 117.0)	99.0 (91.0, 113.0)	0.67
Glycated haemoglobin, HbA1c (%)	5.4 (5.1, 5.5)	5.2 (5.1, 5.5)	5.4 (5.1, 5.7)	0.072
Total cholesterol (mmol/L)	5.1 (4.4, 6.0)	5.0 (4.4, 5.5)	5.3 (4.2, 6.1)	0.35
High density lipoprotein (HDL) (mmol/L)	1.5 (1.3, 1.9)	1.5 (1.3, 1.8)	1.6 (1.4, 2.0)	0.085
Low density lipoprotein (LDL) (mmol/L)	3.0 (2.3, 3.6)	2.9 (2.6, 3.5)	3.1 (2.2, 3.6)	0.94
Triglycerides (mmol/L)	1.0 (0.7, 1.4)	1.0 (0.7, 1.5)	1.0 (0.7, 1.4)	0.93
**Thromboelastography (TEG)**				
R-time (min)	6.3 (5.4, 7.9)	6.3 (5.4, 7.2)	6.5 (5.6, 8.2)	0.30
K-time (min)	2.2 (1.8, 2.6)	2.1 (1.6, 2.4)	2.3 (1.8, 2.8)	0.49
a-angle (°)	58.1 (48.1, 64.9)	58.5 (49.4, 65.0)	57.6 (46.9, 63.4)	0.70
Maximum Amplitude (mm), mean (SD)	59.9 (6.1)	62.0 (5.5)	57.4 (5.9)	**<0.001**
Lysis 30 (%)	0.6 (0.0, 1.5)	0.6 (0.1, 1.3)	0.6 (0.0, 2.6)	0.61
**Calibrated automated Thrombogram (CAT)**				
Lag time (min)	3.1 (2.7, 3.7)	3.1 (2.7, 3.6)	3.3 (2.7, 3.7)	0.41
Endogenous thrombin potential (nM.min)	1349.0 (1176.0, 1552.0)	1360.1 (1180.8, 1528.2)	1336.0 (1151.5, 1590.0)	0.83
Peak thrombin (nM)	227.0 (177.0, 272.3)	233.5 (177.2, 273.7)	221.7 (174.3, 271.1)	0.45
Velocity Index (nM/min)	68.7 (46.2, 99.9)	74.1 (46.2, 100.2)	68.4 (46.6, 99.1)	0.81
**Overall Haemostasis Potential (OHP)**				
Overall coagulation profile, mean (SD)	36.1 (9.8)	34.3 (9.7)	37.8 (9.6)	0.052
Overall haemostasis potential (OHP)	6.5 (4.8, 9.7)	6.2 (4.7, 9.5)	6.7 (5.2, 9.9)	0.30
Overall fibrinolytic potential (OFP) (%)	80.9 (77.2, 84.2)	80.5 (76.9, 84.2)	81.3 (78.3, 84.2)	0.51
**Tissue factor pathway inhibitor** (ng/mL)	12.7 (66.5, 26.7)	10.4 (6.5, 24.1)	16.6 (6.7, 27.6)	0.30

Data reported as median (IQR) unless otherwise stated; Abbreviation: SD standard deviation; APTT activated partial thromboplastin time; eGFR estimated glomerular filtration rate. The assays and significant *p*-values are highlighted in bold.

**Table 2:** Healthy controls compared to patients with cardiovascular risk factors/disease.

Factor	Healthy Controls	Patients with Cardiovascular Risk Factors and/or Disease	*p*-Value
N	123	258	
Age, mean (SD)	41.5 (16.6)	64.5 (13.9)	**<0.001**
Sex (male)	43 (35.0%)	142 (55%)	**<0.001**
**Plasma ACE2 activity** (pmol/mL/min)	0.10 (0.02, 3.33)	5.99 (1.95, 10.37)	**<0.001**
**Comorbidities**			
Obesity, *n* (%)	Data not collected	136 (52.7%)	
Hypertension, *n* (%)	0	212 (82.2%)	
Diabetes mellitus, *n* (%)	0	156 (60.5%)	
Cerebrovascular accident, *n* (%)	0	16 (6.2%)	
Peripheral artery disease, *n* (%)	0	18 (7.0%)	
Coronary artery disease, *n* (%)	0	72 (27.9%)	
Active smoker, *n* (%)	0	45 (17.4%)	
Number of cardiovascular risk factors, mean (SD)	0	4.5 (1.5)	
**Medications**			
Aspirin, *n* (%)	0	106 (41.1%)	
Statin, *n* (%)	0	170 (65.9%)	
ACE inhibitor or ARB, *n* (%)	0	143 (55.4%)	
**Biochemical investigations**			
Haemoglobin (g/dL)	142.0 (136.0, 153.0)	135.5 (119.0, 147.0)	**<0.001**
White cell count (×10^9^/L)	5.7 (5.0, 6.6)	7.3 (6.2, 9.0)	**<0.001**
Platelets (×10^9^/L)	245.0 (215.0, 279.0)	249.5 (195.8, 290.1)	0.82
Prothrombin time (s)	11.0 (10.3, 12.6)	12.0 (10.9, 12.8)	**<0.001**
Activated partial thromboplastin time (s)	28.0 (26.0, 31.0)	28.1 (26.5, 29.9)	0.84
Fibrinogen (g/L)	3.0 (2.5, 3.5)	3.9 (3.3, 4.6)	**<0.001**
D-dimer (mg/L FEU)	270.0 (150.0, 290.0)	570.0 (340.0, 980.0)	**<0.001**
Factor VIII level (%)	104.0 (86.0, 145.0)	161.0 (118.5, 198.0)	**<0.001**
Von Willebrand factor antigen (%)	100.0 (86.0, 127.0)	154.0 (112.0, 193.3)	**<0.001**
Estimated glomerular filtration rate (mL/min/1.73 m^2^)	99.5 (90.0, 116.0)	67.0 (30.0, 90.0)	**<0.001**
Glycated haemoglobin, HbA1c (%)	5.4 (5.1, 5.5)	6.4 (5.5, 7.6)	**<0.001**
Total Cholesterol (mmol/L)	5.1 (4.4, 6.0)	4.3 (3.6, 5.1)	**<0.001**
High density lipoprotein (HDL) (mmol/L)	1.5 (1.3, 1.9)	1.1 (0.9, 1.4)	**<0.001**
Low density lipoprotein (LDL) (mmol/L)	3.0 (2.3, 3.6)	2.2 (1.6, 2.9)	**<0.001**
Triglycerides (mmol/L)	1.0 (0.7, 1.4)	1.8 (1.2, 2.5)	**<0.001**
**Thromboelastography (TEG)**			
R-time (min)	6.3 (5.4, 7.9)	6.2 (5.3, 7.2)	**0.050**
K-time (min)	2.2 (1.8, 2.6)	1.8 (1.4, 2.1)	**<0.001**
a-angle (°)	58.1 (48.1, 64.9)	59.2 (47.8, 68.0)	0.29
Maximum Amplitude, mean (SD)	59.9 (6.1)	69.2 (5.9)	**<0.001**
Lysis 30 (%)	0.6 (0.0, 1.5)	0.1 (0.0, 0.6)	**<0.001**
**Calibrated automated thrombogram (CAT)**			
Lag time (min)	3.1 (2.7, 3.7)	4.0 (3.4, 4.7)	**<0.001**
Endogenous thrombin potential (nM.min)	1349.0 (1176.0, 1552.0)	1310.9 (1132.4, 1482.6)	0.17
Peak thrombin (nM)	227.0 (177.0, 272.3)	221.5 (178.7, 264.9)	0.87
Velocity Index (nM/min)	68.7 (46.2, 99.9)	64.3 (42.3, 92.7)	0.63
**Overall haemostasis potential (OHP)**			
Overall coagulation profile, mean (SD) (unit)	36.1 (9.8)	42.2 (10.5)	**<0.001**
Overall haemostasis potential (OHP)	6.5 (4.8, 9.7)	9.8 (7.0, 14.4)	**<0.001**
Overall fibrinolytic potential (OFP) (%)	80.9 (77.2, 84.2)	74.8 (68.9, 80.2)	**<0.001**
**Tissue factor pathway inhibitor** (ng/mL)	12.7 (66.5, 26.7)	35.2 (17.8, 55.4)	**<0.001**

Data reported as median (IQR) unless otherwise stated; Abbreviation: ACE = angiotensin converting enzyme, ARB = angiotensin receptor blocker, CV = cardiovascular; CVD = cardiovascular disease; SD = standard deviation. The assays and significant p-values are highlighted in bold.

**Table 3:** Plasma ACE2 activity levels reported in tertiles according to cardiovascular risk factors and/or disease and baseline investigations.

	Plasma ACE2 Activity ≤3 pmol/mL/min	Plasma ACE2 Activity 3.1 to 8.2 pmol/mL/min	Plasma ACE2 Activity ≥8.3 pmol/mL/min	*p*-Value ^1^	*p*-Value ^2^
N	85	86	87		
Age, mean (SD)	60.5 (15.9)	64.9 (12.7)	68.2 (12.0)	**<0.001**	
Sex (male)	35 (41%)	46 (54%)	61 (70%)	**0.002**	
**Clinical characteristics**					
Body mass index (kg/m^2^), mean (SD)	30.8 (8.3)	31.7 (7.9)	31.8 (7.8)	0.65	
Systolic blood pressure (mmHg), mean (SD)	133 (16)	136 (18)	136 (20)	0.54	
Diastolic blood pressure (mmHg), mean (SD)	77 (11)	76 (12)	75 (13)	0.69	
Framingham Grade				**<0.001**	**<0.001**
Low (<10)	24 (28.2%)	9 (10.4%)	8 (9.2%)		
Moderate (10–20)	23 (27.1%)	29 (33.7%)	12 (13.8%)		
High (>20)	38 (44.7%)	48 (55.8%)	67 (77.0%)		
**Comorbidities**					
Obesity	38 (44.7%)	50 (58.1%)	48 (55.2%)	0.18	
Hypertension	70 (82.4%)	73 (84.9%)	69 (79.3%)	0.63	
Diabetes mellitus	48 (56.5%)	48 (55.8%)	60 (69.0%)	0.14	
Cerebrovascular accident	4 (4.8%)	7 (8.1%)	5 (5.7%)	0.64	
Peripheral artery disease	3 (3.5%)	5 (5.8%)	10 (11.5%)	0.11	
Coronary artery disease	15 (17.6%)	24 (27.9%)	33 (37.9%)	**0.012**	**0.005**
Active smoker	19 (22.4%)	13 (15.1%)	13 (14.9%)	0.35	
Number of cardiovascular risk factors ^3^, mean (SD)	4.1 (1.7)	4.4 (1.3)	4.8 (1.2)	**0.003**	
**Medications**					
Aspirin	26 (30.6%)	38 (44.2%)	42 (48.3%)	**0.048**	
Statin	53 (62.4%)	58 (68.2%)	59 (67.8%)	0.66	
ACE inhibitor or ARB	42 (49.4%)	49 (57.0%)	52 (59.8%)	0.72	
**Biochemical Investigations**					
Haemoglobin (g/dL)	133.5 (118.8, 145.3)	135.5 (118.8, 148.3)	136.0 (120.0, 147.3)	0.61	
White cell count (×10^9^/L)	7.8 (6.1, 9.1)	7.4 (6.3, 8.8)	7.2 (5.9, 9.0)	0.75	
Platelets (×10^9^/L)	264.5 (198.5, 313.8)	246.5 (197.0, 288.5)	229.0 (171.8, 288.3)	0.063	
Prothrombin time (s)	11.4 (10.7, 12.8)	12.1 (11.0, 12.5)	12.3 (11.4, 13.2)	**0.001**	**0.044**
Activated partial thromboplastin time (s)	28.4 (26.2, 29.5)	27.9 (26.7, 30.1)	28.1 (26.4, 30.4)	0.53	
Fibrinogen (g/L)	3.9 (3.2, 4.5)	4.2 (3.4, 4.9)	3.7 (3.2, 4.5)	0.16	
D-dimer (mg/L FEU)	540.0 (300.0, 955.0)	520.0 (320.0, 840.0)	610.0 (340.0, 1030.0)	0.42	
Factor VIII level (%)	153.5 (114.0, 195.0)	158.5 (114.0, 188.0)	174.5 (130.0, 207.3)	0.072	
Von Willebrand factor antigen (%)	150.0 (110.0, 183.0)	147.0 (102.5, 194.0)	169.0 (122.0, 216.0)	0.096	
C-reactive protein (mg/L)	3.0 (1.0, 8.5)	2.9 (1.3, 6.8)	3.2 (1.2, 6.4)	0.99	
Estimated glomerular filtration rate (mL/min/1.73 m^2^)	65.0 (19.0, 93.5)	72.0 (38.0, 91.0)	61.0 (30.5, 87.0)	0.66	
Glycated haemoglobin, HbA1c(%)	6.1 (5.4, 7.6)	6.3 (5.5, 7.6)	6.4 (5.7, 7.8)	0.18	
Total cholesterol (mmol/L)	4.4 (3.8, 5.4)	4.3 (3.5, 5.0)	4.1 (3.4, 5.0)	0.13	
High density lipoprotein (HDL) (mmol/L)	1.2 (0.9, 1.5)	1.1 (0.9, 1.5)	1.1 (1.0, 1.4)	0.87	
Low density lipoprotein (LDL) (mmol/L)	2.5 (1.8, 3.2)	2.2 (1.5, 2.8)	2.0 (1.6, 2.7)	0.051	
Triglycerides (mmol/L)	1.6 (1.2, 2.4)	1.7 (1.2, 2.4)	1.9 (1.2, 2.7)	0.72	

Values reported as median (IQR) unless otherwise stated. ^1^ *p*-value comparing across the ACE2 activity tertiles; ^2^ *p*-value after age and gender adjustment; ^3^ cardiovascular risk factors include: age > 65 years, male gender, hypertension, hyperlipidaemia, obesity (Body mass index > 30), smoker, family history of cardiovascular disease, severe or end stage chronic kidney disease (eGFR < 30 mL/min/1.73 m^2^). The assays and significant p-values are highlighted in bold.

**Table 4:** Association between global coagulation assays and plasma ACE2 activity levels, reported in tertiles, in patients with cardiovascular risk factors and/or disease.

	Plasma ACE2 Activity ≤3 pmol/mL/min	Plasma ACE2 Activity 3.1 to 8.2 pmol/mL/min	Plasma ACE2 Activity ≥8.3 pmol/mL/min	*p*-Value ^1^	*p*-Value ^2^
N	85	86	87		
**Thromboelastography**					
R-time (min)	6.2 (5.3, 7.6)	6.1 (5.2, 7.5)	6.2 (5.4, 7.0)	0.99	
K-time (min)	1.8 (1.3, 2.2)	1.8 (1.4, 2.2)	1.7 (1.4, 2.1)	0.95	
a-angle (°)	56.4 (46.3, 67.9)	54.0 (46.6, 68.2)	62.4 (50.3, 69.1)	0.28	
Maximum Amplitude (mm), mean (SD)	69.2 (5.6)	68.7 (5.6)	66.1 (6.2)	**0.009**	**0.043**
Lysis 30 (%)	0.1 (0.0, 0.9)	0.1 (0.0, 0.7)	0.1 (0.0, 0.5)	0.70	
**Calibrated Automated Thrombogram**					
Lag time (min)	4.0 (3.4, 4.7)	3.7 (3.3, 4.3)	4.3 (3.7, 5.2)	**0.025**	0.094
Endogenous thrombin potential (nM.min)	1272.2 (1148.1, 1449.6)	1307.5 (1123.2, 1525.2)	1305.1 (1080.5, 1471.1)	0.87	
Peak thrombin (nM)	217.0 (176.3, 254.6)	225.2 (177.1, 296.4)	227.8 (182.3, 260.6)	0.55	
Velocity Index (nM/min)	63.1 (41.4, 85.5)	67.6 (47.7, 110.1)	64.4 (42.4, 93.6)	0.25	
**Overall Haemostasis Potential**					
Overall coagulation profile, mean (SD) (unit)	42.8 (11.8)	42.1 (9.9)	38.9 (9.5)	**0.035**	0.086
Overall haemostasis potential (OHP)	10.7 (6.5, 15.6)	10.5 (7.6, 14.6)	9.2 (6.6, 12.7)	0.51	
Overall fibrinolytic potential (OFP) (%)	75.2 (67.7, 80.4)	74.7 (67.6, 79.6)	74.9 (68.8, 81.2)	0.95	
**Tissue factor pathway inhibitor** (ng/mL)	37.4 (19.1, 56.7)	26.9 (13.5, 46.3)	36.3 (17.1, 77.1)	**0.027**	**0.044**

Values reported as median (IQR) unless otherwise stated. ^1^ *p*-value comparing across the ACE2 activity tertiles; ^2^ *p*-value after age and gender adjustment. The assays and significant *p*-values are highlighted in bold.

## Data Availability

Not applicable.
